# An analysis of companion animal tick encounters as revealed by photograph‐based crowdsourced data

**DOI:** 10.1002/vms3.586

**Published:** 2021-08-20

**Authors:** Heather L. Kopsco, Roland J. Duhaime, Thomas N. Mather

**Affiliations:** ^1^ Center for Vector‐Borne Disease, University of Rhode Island Kingston Rhode Island; ^2^ TickEncounter Resource Center Kingston Rhode Island; ^3^ Department of Pathobiology College of Veterinary Medicine University of Illinois Urbana‐Champaign Urbana Illinois; ^4^ Environmental Data Center, University of Rhode Island Kingston Rhode Island

**Keywords:** cats, community science, dogs, tick‐borne disease

## Abstract

**Background:**

Community science is increasingly utilized to track important vectors of companion animal disease, providing a scalable, cost‐effective strategy for identifying new foci, changing phenology, and disease prevalence across wide geographies.

**Objectives:**

We examined photographs of ticks found attached to predominately dogs and cats reported to a photograph‐based tick surveillance program to identify potential areas for improvements in tick prevention education and risk intervention.

**Methods:**

We compared estimated days of tick attachment using a Kruskal–Wallis one‐way analysis of variance, and a Pearson's chi‐square analysis of variance on the number of submissions by host type submitted for each season.

**Results:**

The blacklegged tick (*Ixodes scapularis*) was the most common species reported (39.8%). Tick photographs submitted were almost entirely adults (89.5%), and ticks found on companion animals exhibited an estimated median engorgement time of 2.5 days. *Ixodes scapularis* displayed the highest median engorgement of the top tick species found feeding on companion animals (*χ* ^2^ = 98.96, *p* < 0.001). Ticks were spotted year‐round; during spring and summer, ticks collected from pets represented 15.4 and 12.8% of all submissions, but increased to 28.5 and 35.2% during autumn and winter, respectively.

**Conclusions:**

Crowdsourced data reveal that mostly adult ticks are detected on pets, and they are found at a point in the blood‐feeding process that puts pets at heightened risk for disease transmission. The increase in proportion of ticks found on pets during colder months may reveal a critical knowledge gap amongst pet owners regarding seasonal activity of *I. scapularis*, a vector of Lyme disease, providing an opportunity for prevention‐education.

## Graphical abstract

Crowdsourced data reveal that mostly adult ticks are spotted on pets, and that they are detected later in the blood‐feeding process than were ticks attached to humans, putting pets at heightened risk for tick‐borne disease transmission. The doubling of reports of ticks found on pets during autumn and winter may reveal a critical knowledge gap amongst the pet‐owning general public regarding seasonal activity of *I. scapularis*, vector of Lyme disease, providing an opportunity for prevention‐education to pet owners.

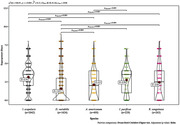

## 1 INTRODUCTION

Tick vectors can pose serious health risks to domestic companion animals and their owners. Dogs, cats and other domestic animals are susceptible to numerous potentially deadly tick‐borne bacterial and protozoal diseases, including Lyme disease, spotted fever rickettsioses, American canine hepatozoonosis, cytauxzoonosis and tick paralysis toxicosis (Bowman 2009; Chomel 2011; Kidd & Breitschwerdt 2003; Little et al., 2014; Nagamori & Reichard 2015; Reichard et al., 2010; Shaw et al., 2001). Even in cases where animals are asymptomatic or display sub‐clinical signs of tick‐borne disease infection, they can act as potential reservoirs for zoonotic pathogens (Fritz 2009; Mather et al., 1994; Shannon et al., 2017; Shaw et al., 2001). Several studies have demonstrated that those living in households with either dogs or cats are at higher risk of tick encounters and tick‐borne illnesses than those in households without pets (de Wet et al., 2020; Jones et al., 2002, 2018). Cats that live entirely indoors have been found infested with species of attached ticks that originate in outdoor habitats (Little et al., 2018). This finding suggests that cats, and perhaps even dogs or humans can carry loose ticks indoors that then attach to other hosts. The strong association among pets, their owners and zoonotic tick‐borne illness is evident in the literature, as dogs often act as sentinels for tick‐borne illness, particularly in cases of Rocky Mountain Spotted Fever (Elchos & Goddard 2003), and Lyme disease (Bowman et al., 2009; Eng et al., 1988; Guerra et al., 2001; Johnson et al., 2004; Lindenmayer et al., 1991; Little et al., 2014; Wagner & Erb 2012).

Recent reports suggest that cases of human tick‐borne disease are more than double over the past decade (Rosenberg et al., 2018), no doubt also increasing significantly the tick‐borne disease prevention challenges faced by pet owners and veterinary professionals. The factors contributing to more cases of tick‐borne diseases are complex. Broad ecological changes, including alterations in climate and land‐use patterns (Allan et al., 2003; Brownstein et al., 2005; Gilliam et al., 2018; Guerra et al., 2002; Hall et al., 2002; Ostfeld & Brunner 2015; Salkeld et al., 2015; Tran & Waller 2013), and increases in deer and other wildlife populations overlapping with human‐inhabited space, are highly associated with increases in tick encounters and tick‐borne disease prevalence (Ginsberg & Zhioua 1999; Rand et al., 2003; White & Gaff 2018). Additionally, changes in human behaviour resulting in enhanced exposure risks (Fischhoff et al., 2019; Zeimes et al., 2014), and overall improvements in disease diagnosis, surveillance and reporting (Rosenberg et al., 2018) are also responsible for higher occurrence of disease. Concurrently, distributions of tick species are changing, especially blacklegged ticks (*Ixodes scapularis* Say) and lone star ticks (*Amblyomma americanum* L.) (Dahlgren et al., 2016; Eisen et al., 2016; Springer et al., 2014), exacerbating the problem while changing climate trends can alter the timing and length of tick and host activity periods, as well as the length of the overlap in companion animal outdoor activity in tick habitat (Alkishe et al., 2021; Ogden & Lindsay 2016; Ogden et al., 2014). Redistribution of wildlife, especially cervids, such as white‐tail deer, and movement of ticks on migrating birds, is resulting in the incursion of ticks into more densely populated suburban, peri‐urban and even urban areas (Shaw et al., 2001). As a result, new endemic foci (Schwartz et al., 2017; Sonenshine 2018) are putting a larger demographic of humans and pets at risk for tick attachment and illness.

**IMPACTS**

- The blacklegged (deer) tick was the most commonly reported tick attached to companion animals.
- Ticks found attached to companion animals have been fed a median of 2.5 days longer than compared to those found attached to humans. The blacklegged (deer) tick had the highest median engorgement of the five top tick species found feeding on companion animals.
- The percentage of reports of ticks found on companion animals is more than doubled in the fall and winter seasons from what was reported in the spring and summer seasons.

Passive surveillance, particularly involving the contributions of community science, has become an increasingly popular method for tracking ticks on pets and associated pathogens. Often, ticks are collected from pet owners at veterinary offices or other research centres (Johnson et al., 2004; Nieto et al., 2018; Saleh et al., 2019; Xu et al., 2016), identified to species, and tested for a wide range of pathogens. These large‐scale collection efforts can be examined over time to observe trends in tick distributions and infection rates (Saleh et al., 2019; Sánchez‐Vizcaíno et al., 2016; Tulloch et al., 2017). Ventures using big data, including internet search terms, social media and electronic health records, are also showing promise as a means of tracking tick‐borne disease in companion animals in an increasingly digital world (Guernier et al., 2016; Tulloch et al., 2019).

Photographs of encountered ticks have been shown to be another reliable method of rapid identification of commonly encountered ticks of medical concern (Fernandez et al., 2019; Koffi et al., 2017; Kopsco et al., 2020) that can be used to not only track tick trends, but also relay important information about potential disease risk to pet owners or veterinarians. The TickSpotters program at the University of Rhode Island's Tick Encounter Resource Centre (TERC) is an online, photograph‐based passive surveillance system, staffed by PhD and other graduate‐level tick researchers, with an overall accuracy of 96.7% for North American ticks most commonly encountered by humans and their companion animals, particularly *I. scapularis, Dermacentor variabilis*, and *A. americanum* (Kopsco et al., 2020). These three tick species are the most commonly encountered tick species by dogs and cats throughout the United States (Duncan et al., 2021; Ghosh et al., 2021; Saleh et al., 2019). The system allows users to submit photographs of ticks along with information surrounding each encounter. Moreover, a response email is sent within 24‐h that confirms the identity of the tick to species and stage, estimates a duration window of attachment, and provides tailored guidance regarding likely riskiness for infection and best next actions to prevent disease and avoid future tick encounters.

The purpose of this study was to examine photographs of ticks found attached to pets and reported to TickSpotters photograph‐based tick surveillance to identify potential areas for improvements in tick prevention education and risk intervention. We anticipated that photographed tick submissions from pets would exhibit unique characteristics that differed from submissions of ticks found on humans. Specifically, we expected that proportionally more ticks would be detected on pets than on humans during the fall and winter seasons when owners potentially assume that ticks are no longer an active concern, and that ticks attached to pets would be found later in the feeding process (i.e., would display engorgement indicative of a longer feeding duration) than ticks reported from humans.

## 2 METHODS

### 2.1 Data collection

We performed a retrospective analysis on data collected through the TickSpotters photograph‐based crowdsourced surveillance program (Wufoo online forms, SurveyMonkey, Inc.) from January 1, 2014 to December 31st, 2018 to describe a sample of domestic animal tick encounters as reported by their owners or veterinarians, and to compare key aspects of these animal tick encounters to those experienced by humans. The survey was advertised on a weekly basis through the TERC social media pages (Facebook and Twitter), and participants were directed to submit details of their tick encounter along with a photograph on the submission website (https://tickencounter.org/tickspotters/submit_form). Prior to uploading a photograph of the specimen to the system, instructions were provided to participants on how to take a photograph of the specimen with a size reference, proper lighting and clear focus to ensure the image highlighting the necessary anatomy to facilitate correct identification by researchers. Submissions with pictures that could not be identified by TERC researchers were recorded as ‘Unknown’, and a request was sent for an additional improved photograph. Records were accordingly updated with an identification if a better picture was sent in response to this request. TERC medical entomologists and staff trained in tick identification reviewed entries daily, and tick photographs were identified to species, stage and an attachment duration estimate. A visual estimated tick feeding duration chart (https://web.uri.edu/tickencounter/fieldguide/tick‐growth‐comparison‐charts/) was previously established for the most commonly encountered tick species and their life stages by feeding groups of five or more ticks of each species and stage featured in the TERC pictorial chart on New Zealand white rabbits, and removing the ticks at specified time points (24, 36, 48 h, etc.). A scutal index (ratio of scutum width to body length) was calculated for each fed tick based on previous methods performed for *I. scapularis* ticks (Falco et al., 2018; Yeh et al., 1995), and the tick demonstrating the median size was photographed as representative for that time point (Mather, unpublished data). Tick photographs were examined against the TERC pictorial chart displaying different species at half‐day fed intervals and the closest estimated feeding window was assigned. Feeding duration of tick species that did not have a representative image was compared to the life stage of the other validated tick species and a 24‐h feeding window was estimated. A tailored email response was sent to the participant with species identification confirmation and a riskiness assessment based on the tick/stage‐specific diseases and tick infection estimates of those diseases derived from published studies if available for the region where the tick was encountered. Information also was provided in the email on how to prevent future tick encounters and attachments by conducting regular tick checks, using permethrin repellent and various tick ‘knock‐down’ pet products (i.e., products where ticks are not only repelled but by which are also quickly immobilized and killed), and resources to submit ticks for testing should concern exist regarding infection. The collection survey was approved and overseen by the University of Rhode Island Institutional Review Board (# HU1819‐092).

### 2.2 Statistics

We calculated basic descriptive statistics and proportions to understand the relative contributions of different tick species and feeding times to the overall submission of ticks from companion animals. To determine whether there were significant differences in duration of attachment (based on an engorgement index) among tick species, we compared estimated days of attachment using a Kruskal–Wallis one‐way analysis of variance due to unequal variances and non‐normal distribution of the tick feeding times (Shapiro–Wilk Normality Test for Engorgement (Days): *p*‐value = < 0.001, and Bartlett's test for homogeneity of variances for factor Species: *p*‐value = < 0.001). Post‐hoc pairwise analysis using the Dwass—Steel–Crichtlow–Fligner method and adjusted *p*‐values was subsequently conducted to identify differences between species. We also performed a Pearson's chi‐square analysis of variance on the number of tick submissions by host type submitted for each season (spring, summer, fall winter). Effect sizes to understand the magnitude of the significant differences for both of these analyses were calculated (epsilon‐squared and Cramer's V, respectively) and listed along with significant *p*‐values and confidence intervals (Rea & Parker 1992).

## 3 RESULTS

From January 1, 2014 to December 31st, 2018 TickSpotters received 31,684 photo submissions from throughout the United States and several other countries. Of those reported from the United States (n = 29,528), 17.3% (n = 5,132) of submissions were specimens removed from domestic animals stated or assumed to be pets, while 72.4% (n = 21,366) were reportedly found on human hosts (Kopsco et al., 2021), and 10.3% (n = 3,030) were found unattached to a host (i.e., loose and wandering). Over 70% of the specimens reported from pets came from Northeastern (Maine, New Hampshire, Vermont, Massachusetts, Rhode Island), Mid‐Atlantic (Connecticut, Delaware, District of Columbia, Maryland, New York, New Jersey, Pennsylvania, Virginia) and midwestern (Illinois, Indiana, Iowa, Kansas, Michigan, Minnesota, Missouri, Nebraska, North Dakota, Ohio, South Dakota, Wisconsin) states, with less than 20% of submissions coming from each of the other regions of the country (Table 1). Roughly half (53.5%) of submissions came from states considered endemic for Lyme disease (CT, DE, MA, MD, ME, MN, NH, NJ, NY, PA, RI, VA, VT, WI) (i.e., the 14 states reporting 95% of human Lyme disease cases as of 2015) (CDC 2020; Diuk‐Wasser et al., 2012; Schwartz et al., 2017) (Table 1). Reports were of specimens largely found on pet dogs (45.7%), however, nearly half of submissions did not specify either dog or cat because this information was not split when collected by the submission form until the end of 2017. Participants noted when ticks were found on an animal that was neither a dog nor a cat, for example, a companion horse, or in the case of wildlife encountered in a wildlife clinic setting.

**TABLE 1:** Distribution of confirmed TickSpotters submissions from pets, 2014 to 2018 (n = 5132)

Species	n = 5132 (%)
Blacklegged tick (*Ixodes scapularis*)	2044 (39.8)
American dog tick (*Dermacentor variabilis*)	1625 (31.6)
Lone star tick (*Amblyomma americanum*)	400 (7.8)
Brown dog tick *(Rhipicephalus sanguineus sensu lato*)	263 (5.1)
Western‐blacklegged tick (*Ixodes pacificus*)	230 (4.5)
Unknown tick species	207 (4.0)
Rocky Mountain wood tick (*Dermacentor andersoni*)	148 (2.9)
Gulf coast tick (*Amblyomma maculatum*)	65 (1.3)
Not a tick	59 (1.1)
Pacific coast tick (*Dermacentor occidentalis*)	53 (1.0)
*Ixodes angustus*	15 (0.3)
Rabbit tick (*Haemaphysalis leporispalustris*)	7 (0.1)
Winter tick (*Dermacentor albipictus*)	4 (0.07)
Soft tick (*Argasidae* sp.)	3 (0.06)
East Asian longhorned tick (*Haemaphysalis longicornis*)	2 (0.04)
Spinose ear tick (*Otobious megnini*)	2 (0.04)
Woodchuck tick (*Ixodes cookei*)	2 (0.04)
Raccoon tick (*Ixodes texanus)*	1 (0.02)
*Amblyomma* spp.	1 (0.02)
Poultry tick (*Argas persicus*)	1 (0.02)

Life stage	n = 5132 (%)
Adult	4594 (89.5)
Nymph	207 (4.03)
Larva	52 (1.01)
Unknown	279 (5.43)

Tick engorgement estimate	n = 5033 (%)
Less than one day	1781 (35.4)
1‐2 days	698 (13.9)
2.5‐3.5 days	1065 (21.2)
4‐5 days	1194 (23.7)
5.5‐7.5	276 (5.5)
>7.5 days	19 (0.38)

Region	n = 5132 (%)
New England (CT, ME, MA, NH, RI, VT)	945 (18.4)
Middle Atlantic (NJ, NY, PA)	1190 (23.2)
South Atlantic (DC, DE, FL, GA, MD, NC, SC, VA, WV)	559 (10.9)
East South Central (AL, KY, MS, TN)	162 (3.2)
West South Central (AR, LA, OK, TX)	222 (4.4)
East North Central (IL, IN, MI, OH, WI)	953 (18.6)
West North Central (IA, KS, MN, MO, ND, NE, SD)	196 (3.8)
Mountain (AZ, CO, ID, MT, NM, NV, UT, WY)	235 (4.6)
Pacific (AK, CA, HI, OR, WA)	670 (12.9)

Lyme endemicity	n = 5132 (%)
Endemic states (CT, DE, MA, MD, ME, MN, NH, NJ, NY, PA, RI, VA, VT, WI)	2746 (53.5)
Non‐endemic states	2386 (46.5)

Animal type*	n = 5132 (%)
Dog	2345 (45.7)
Cat	204 (4.0)
Either dog or cat**	2541 (49.5)

Engorgement estimates were determined by comparison to a tick engorgement gauge based on the scutal index. Lyme endemic states include CT, DE, MA, MD, ME, MN, NH, NJ, NY, PA, RI, VA, VT, WI (Bacon et al., 2008; Diuk‐Wasser et al., 2012; CDC 2020). States are grouped into regions according to the US Census Bureau and Hook et al., 2015. Proportions and totals for tick engorgement are based on the total number of ticks submitted (i.e., minus the ‘Not ticks’ and unknown tick species for which a duration of attachment could not be made).

* A total of 42 (0.8%) submissions were ticks from family livestock companions (e.g., horses) or patients at wildlife clinics.

** Dogs and cats recorded separately beginning in 2017.

**FIGURE 1:**
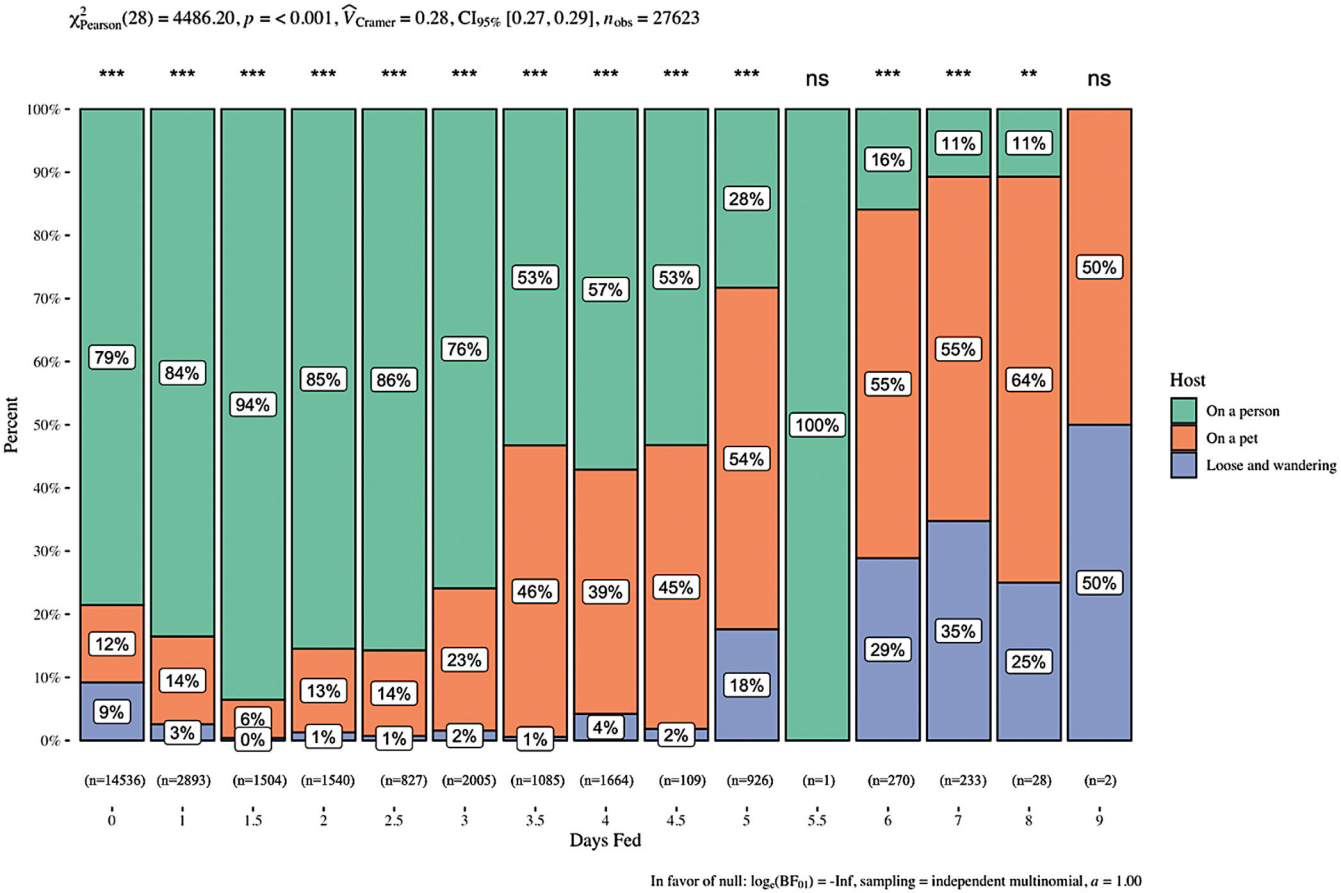
Comparison of estimated tick feeding time (days) on companion animals versus those found on humans or found unattached for ticks reported to the TickSpotters Program from 2014 to 2018. Tick feeding time was assessed by comparison to a pictorial tick engorgement chart based on the scutal index. Median engorgement for human‐encountered ticks (n = 20,710) was 1 day‐fed (SD = 1.39, SE = 0.01, range = 0‐8 days). Median attachment for pet‐encountered ticks (n = 5033) was 2.5‐days‐fed (SD = 2.11, SE = 0.01, range = 0‐9 days). Median engorgement for unattached ticks (unfed or replete) (n = 1880) was 0 days‐fed (SD = 2.29, SE = 0.05, range = 0–9 days). *** *p* < 0.001. ** *p* < 0.01. ns = Not significant

The four most commonly encountered tick species by pets were blacklegged ticks (*I. scapularis*) (40.5%), followed by American dog ticks (*D. variabilis*) (28.2%), lone star ticks (*A. americanum*) (8.2%) and brown dog ticks (*Rhipicephalus sanguineus sensu lato*) (6.5%). Adult stage ticks comprised 90.8% of the submitted tick photographs for pets, and while 35.4% of tick photos submitted showed ticks that had been attached to pets for less than 1 day, more than 50% of ticks were attached for approximately 2.5 days or longer (Table 1). The median engorgement of ticks fed on pets was 2.5 days (standard deviation = 2.11, range 1‐9 days), while the median engorgement of ticks reported from humans was 1 day (standard deviation = 1.39, range 1‐8 days), and those found unattached (either unfed or fed to repletion and detached) also fed for a median of 1 day (standard deviation = 2.29, range = 1‐9 days) (Figure 1). There was a significant difference in tick feeding duration among host type (person, pet, unattached) (*χ* ^2^ = 4486.2, *p* < 0.001), and a large effect size (Cramer's *V* = 0.28, CI [0.27, 0.29] (Figure 1) (Rea & Parker 1992). All tick feeding duration time categories displayed significantly different (*p* < 0.001 or 0.01) proportions of hosts with ticks of that engorgement. Feeding duration estimated at the 5.5 day feeding window was not statistically significant because we only received one tick submission that was estimated to be attached for 5.5 days on a human, and at the 9‐day estimated time because we received only two ticks estimated at approximately 9 days each (one on a pet and one was found on the floor after detaching from an unknown host) (Figure 1).

**FIGURE 2:**
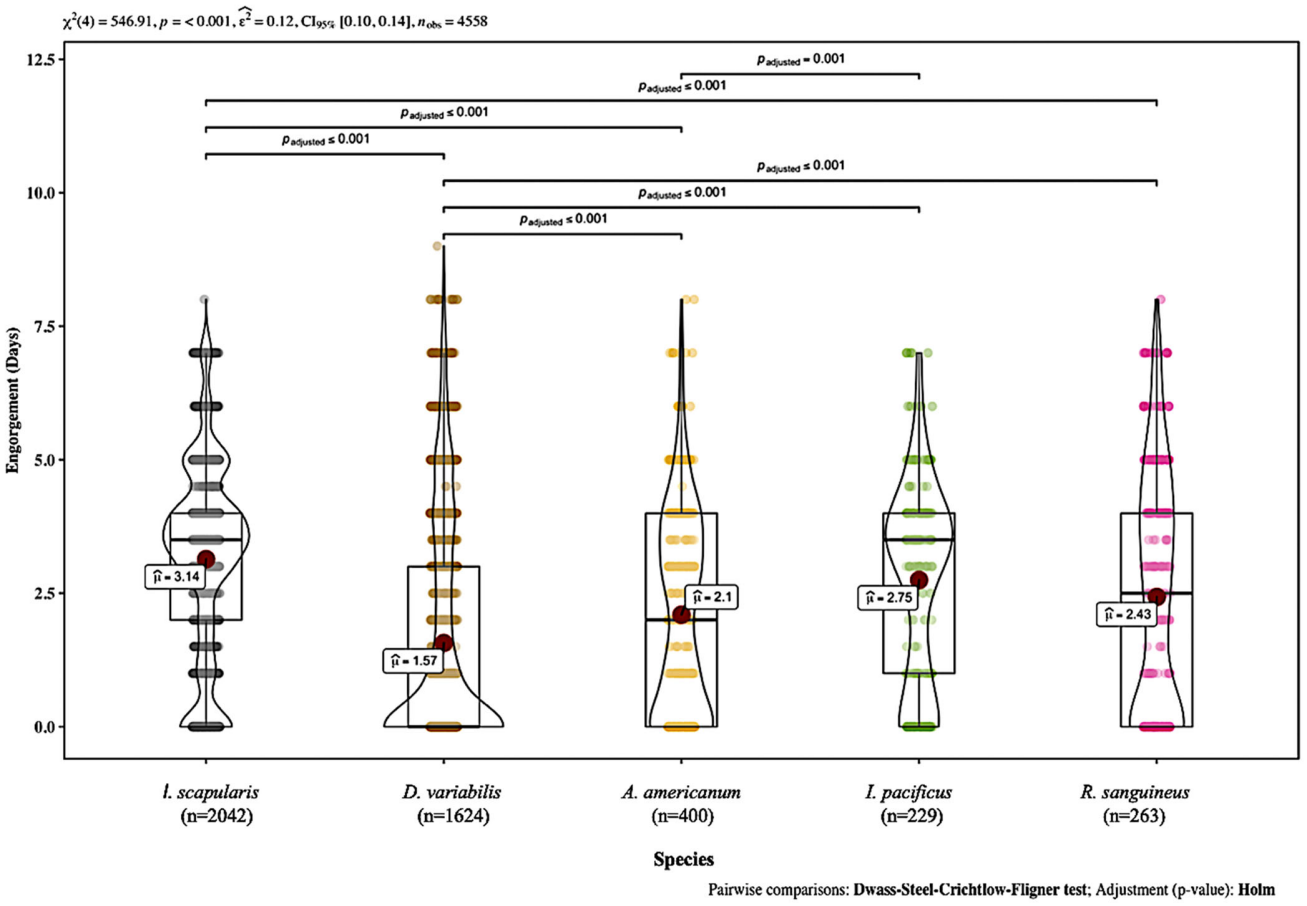
Violin boxplot displaying engorgement at detection (days) of the five most commonly reported tick species found on pets that were submitted to TickSpotters from 2014 to 2018. Feeding times were estimated by comparison to a pictorial tick engorgement chart created by TERC that removed feeding ticks from hosts at half day intervals, and was based on the scutal index. The horizontal bar within the box represents the median engorgement for each species. The dots represent the mean engorgement. Kruskal–Wallis nonparametric analysis of variance tested statistical difference among the species engorgement distributions. Effect size is denoted by episilon^2^. Post‐hoc pairwise comparisons were conducted using the Dwass‐Steel‐Crichtlow‐Fligner method and (Holm) adjusted *p*‐values for statistically significant distributions are noted between species whose overall submissions were statistically different in engorgement

Of the five most commonly submitted tick species, blacklegged ticks (both *I. scapularis* and *Ixodes pacificus*) displayed the highest median attachment duration at the time they were detected on pets based on their engorgement index (3 days), followed by brown dog ticks (2.5 days), lone star ticks (2 days) and American dog ticks (<1 day). There was a highly significant difference among these five species (*χ* ^2^ = 546.91, *p* < 0.001) in their attachment duration, but the effect size, or practical magnitude of this difference was only moderate (*ε* ^2^ = 0.12, CI [0.10‐0.14]) (Figure 2) (Rea & Parker 1992). Post‐hoc pairwise comparisons showed highly significant (*p* < 0.001) differences between attachment durations at detection for each of these tick species when feeding on pets (Figure 2).

**FIGURE 3:**
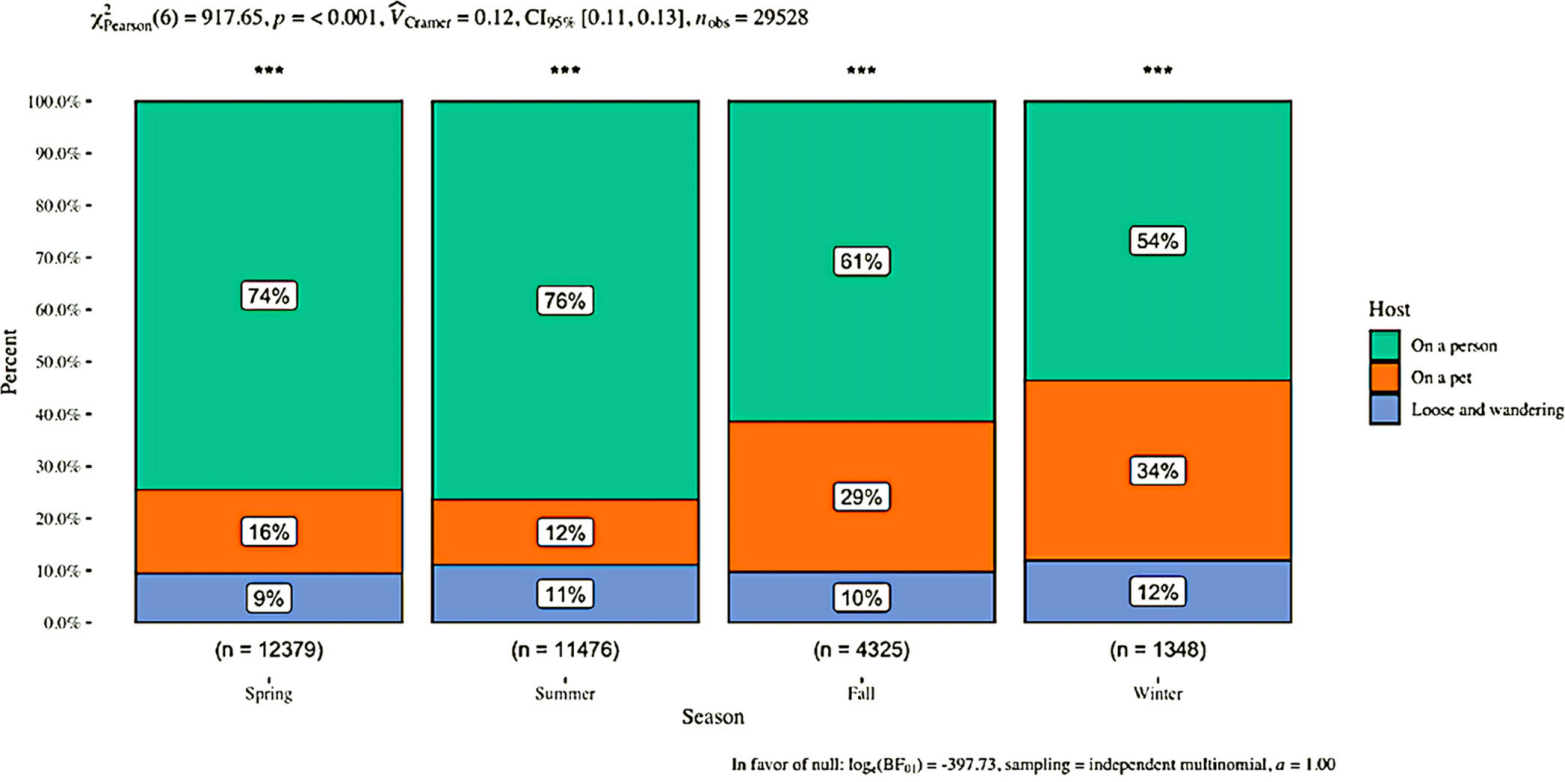
Comparison of seasonal tick encounter submissions by host (i.e., on whom or how the tick was found) out of the total United States submissions (no pet tick encounters were submitted from other countries) using Pearson's chi‐square analysis. Effect size is denoted by Cramer's *V*. ***p < 0.001

The proportion of ticks submitted from pets was significantly different from those found on humans and from tick found unattached in each season (*p* < 0.001), as well as across seasons (*p* < 0.001 (Figure 3). In the spring (March‐May) and summer (June‐August), ticks reported on pets constituted only 16 and 12% of all submissions, respectively, but rose to 29% of all submissions in the fall (September‐November) and 34% of all submissions in the winter months (December‐February) (Figure 3). The proportion of submissions reporting unattached specimens remained relatively constant across months (between 9 and 12% of all submissions) (Figure 3).

## 4 DISCUSSION

We described 5132 domestic companion animal tick encounters reported to a photograph‐based passive tick surveillance system during a 4‐year period. Overall, we found that this group of domestic animals encounters commonly occurring tick species that present a potential disease risk, and also pose a threat to the humans with whom they share a household (Jones et al., 2018). Blacklegged ticks (*I. scapularis*) were the most frequently reported species encountered by companion animals, and the species demonstrating the highest median feeding duration rate of the five most commonly encountered ticks. It is well documented that risk for transmission of tick‐borne pathogens increases with longer duration of tick feeding (des Vignes et al., 2001; Dolan et al., 2017; Eisen 2018; Kidd & Breitschwerdt 2003; Piesman et al., 1987; Sood et al., 1997). The proportion of ticks reported from pets was more than double in the fall and winter months (Figure 3) indicating that there remains a general lack of awareness that colder months still pose a risk for tick encounters. These findings echo similar results from other investigations into ticks reported from pets (Saleh et al., 2019). Taken together, these observations are troubling due to the numerous diseases that *I. scapularis* can vector and transmit to both pets and humans. A recent survey of pet owners found that people whose pets had a tick encounter within the past 6 months were more likely to encounter a tick themselves (de Wet et al., 2020). Opportunities exist to increase awareness on this point among pet owners through education by both veterinarians and public health agencies promoting a One Health focus to tick‐borne disease prevention. We propose below several targeted areas for educational intervention that can potentially address our findings and help protect pet and human health.

It is imperative that the public have knowledge of seasonal tick activity in their locale (Dryden & Payne 2004). Misconceptions regarding which ticks are capable of transmitting which diseases is pervasive (Halperin et al., 2013) and can lead to both undiagnosed disease cases, and falsely believing that one is infected. In particular, Lyme disease is often surrounded in controversy and confusion (Auwaerter et al., 2011). Awareness that *I. scapularis* and *I. pacificus* ticks potentially carrying the Lyme disease agent are still active in the fall and winter would help support year‐round tick prevention in areas where these ticks occur (Dryden & Payne 2004). Our findings emphasize the importance of more broadly publicizing these ticks’ seasonal activity. We recommend that this information be disseminated before and during the fall tick season via veterinary clinics, mass media and through public health social media pages.

Tick checks on pets are important for detecting and removing loose and wandering ticks before attachment, and early on in the feeding process due to transmission delays for pathogens (Dolan et al., 2017; Eisen 2018; Kidd & Breitschwerdt 2003; Piesman et al., 1987; Sood et al., 1997). Pet checks should be conducted routinely, both immediately after outdoor activity and during regular intervals after the pet has come indoors, to catch any ticks that may have been missed in previous checks. Common areas for tick attachment include the head and ears, back, axillary, inguinal, feet and tail/perianal regions (Little et al., 2019; Saleh et al., 2019), and these ticks should be removed as soon as they are found using fine‐tipped tweezers (Jones et al., 2002). While it may be that the higher proportion of ‘found’ ticks on pets than humans during fall and winter is related to fewer ticks successfully attaching to humans due to more clothing barriers, the longer duration of tick attachment on pets compared to humans is suggestive that pets are not being checked regularly or thoroughly enough, or that owners are less inclined to continue use of effective tick prevention products when they believe tick activity is lower.

Effective industry‐tested products, including topical tick preventative (synthetic pyrethroid‐based formulations), systemic medications (isoxazolines) and vaccines (e.g., against Lyme disease‐causing bacterial species) are a necessary part of the tick‐borne disease prevention toolkit in pets (de la Fuente et al., 2015; Littman et al., 2018). Numerous topical products exist but often do not repel or kill all species of ticks, and many require a tick to attach in order to get a lethal dose of the pesticide. We first recommend products that repel and kill or immobilize ticks before attachment, but suggest veterinarians and public health professionals discuss with pet owners their concerns regarding pesticide usages and develop a solution that both placates fears regarding synthetic chemical usage and still keep pets protected (Isman & Grieneisen 2014; Johnson 2018).

Dog and cat owners should be aware that traveling with pets to areas with different tick species can potentially result in tick importation. Passive tick surveillance in the United Kingdom revealed that over the past 10 years, canine travel resulted in the introduction of ten new tick species from 15 different countries, including the importation of *R. sanguineus* from Cyprus and Spain (Abdullah et al., 2016; Hansford et al., 2018). TickSpotters surveillance has spotlighted known ‘hotspots’ of high *R. sanguineus* activity in the south‐western and south‐eastern US, suggesting that pets were not properly protected from tick activity while in these regions. Pet owners and veterinarians need to be mindful of travel to and from these areas so pets can be properly protected and avoid transporting ticks. Pets are capable of transporting the newly invasive Asian longhorned tick (*Haemaphysalis longicornis*) into new foci, as evidenced by dogs being a common host for this species throughout its realized range. Notably, the single occurrence of *H. longicornis* in Arkansas to date was from a dog (Beard et al., 2018). One confirmed TickSpotter report demonstrated pet transport of an adult female *H. longicornis* from New Jersey to Colorado. Movement of even a single engorged female on a pet could potentially establish a new population of *H. longicornis* since this tick currently reproduces parthenogenically in the United States (Chen et al., 2014; Rainey et al., 2018).

Photograph‐based tick surveillance can provide a complementary strategy for broadly monitoring common tick species encounters with domestic companion animals, even on a national scale. In particular, this method can identify important gaps in pet owner behaviour and practices related to tick prevention, and inform and encourage users of the platform about best tick prevention practices through timely, tailored emailed responses. We, however, acknowledge several limitations to this method, including photographic identification of less commonly encountered tick species, precise estimation of tick attachment time and regional sampling bias. The method used to validate tick identification by photograph did not focus specifically on *R. sanguineus* (Kopsco et al., 2020), however, the characteristic shape of the palps and one‐host behaviour of this tick species, with active indoor infestations often reported by participants along with photographs, assisted in confirming identification of this tick. The vast majority of tick species submitted via photographs were identified as either *I. scapularis* or *D. variabilis*. In most cases where less commonly encountered ticks were suspected, we did not request that the participant submit an in‐hand specimen for identification unless the suspected tick seemed out of its expected geographical range or seasonal activity. One exception to this was for suspected *H. longicornis* due to its close similarity to *Haemaphysalis leporispalustris* and the currently standard procedure for confirming this tick by molecular diagnostics or submission to the USDA National Veterinary Services laboratory. Whenever a *Haemaphysalis* spp. tick photograph was submitted, we sent a prepaid envelope to the participant to mail us the specimen so that it could be confirmed by microscopic investigation, and then by molecular sequencing at the USDA National Veterinary Services Laboratory. The method used to estimate tick feeding duration based on the scutal index was only validated for three species and their life stages under laboratory conditions, so we were only able to provide a window of attachment estimates, instead of precise time points. Ticks feeding times can vary to some extent depending on various conditions, including temperature (Pollock et al., 2015) and host/tick infection status (Couret et al., 2017), but a 24‐h window of feeding time provides appropriate coverage of most tick‐borne pathogen transmission windows. However, this estimate may not be sufficient for properly identifying risk if a tick was subjected to interrupted feeding, and pathogen transmission time was expedited (Tahir et al., 2020). We recognize that the north‐eastern and mid‐Atlantic regions of the country were overrepresented in this sample, and therefore, these results cannot be extrapolated to all areas of the United States, and future work should strive to obtain a balanced proportion of submissions from all regions.

In cases where there was a question about photograph quality, additional photographs were requested of both ventral and dorsal sides of the tick. Since the submission of samples was heavily skewed to the northeast and middle Atlantic regions of the United States, the description of which tick most frequently infests companion animals cannot be generalized to, and may not reflect the larger population of pet encounters. Additionally, many of the 3,030 ticks classified as loose and wandering in the TickSpotters surveillance system were fully engorged ticks that could have fallen off of a pet. Since we did not know for certain if there was a pet in the household in those cases, we still provided pet‐tailored prevention information. Ultimately, our investigation identified several important opportunities for prevention‐education to pet owners, and suggests the utility of this type of surveillance for companion animal tick encounters.

## AUTHOR CONTRIBUTIONS

Roland J. Duhaime: Data curation; resources; software; writing‐review & editing. Thomas N. Mather: Conceptualization; data curation; funding acquisition; project administration; supervision; writing‐review & editing.

## CONFLICT OF INTEREST

All authors declare that they have no conflict of interest.

## ETHICAL STATEMENT

The authors confirm that the ethical policies of the journal, as noted on the journal's author guidelines page, have been adhered to. Ethical approval was maintained through the University of Rhode Island's Institutional Review Board.

### PEER REVIEW

The peer review history for this article is available at https://publons.com/publon/10.1002/vms3.586

## Data Availability

The data that support the findings of this study are available from the corresponding author upon reasonable request.

## References

[vms3586-bib-0001] Abdullah, S. , Helps, C. , Tasker, S. , Newbury, H. , & Wall, R. (2016). Ticks infesting domestic dogs in the UK: A large‐scale surveillance programme. Parasites & Vectors, 9(1), 391. 27388169 10.1186/s13071-016-1673-4 PMC4936200

[vms3586-bib-0002] Alkishe, A. , Raghavan, R. K. , & Peterson, A. T. (2021). Likely geographic distributional shifts among medically important tick species and tick‐associated diseases under climate change in North America: A review. Insects, 12(3), 225. 33807736 10.3390/insects12030225 PMC8001278

[vms3586-bib-0003] Allan, B. F. , Keesing, F. , & Ostfeld, R. S. (2003). Effect of forest fragmentation on lyme disease risk. Conservation Biology: The Journal of the Society for Conservation Biology, 17(1), 267–272.

[vms3586-bib-0004] Auwaerter, P. G. , Bakken, J. S. , Dattwyler, R. J. , Dumler, J. S. , Halperin, J. J. , Mcsweegan, E. , Nadelman, R. B. , Connell, S. O. , Shapiro, E. D. , Sood, S. K. , Steere, A. C. , Weinstein, A. , & Wormser, G. P. (2011). Antiscience and ethical concerns associated with advocacy of Lyme disease. Lancet Infectious Diseases, 11(9), 713–719. 21867956 10.1016/S1473-3099(11)70034-2 PMC4489928

[vms3586-bib-1005] Bacon, R. M. , Kugeler, K. J. , Mead, P. S. , & Centers for Disease Control and Prevention (CDC). (2008). Surveillance for Lyme disease–United States, 1992‐2006. Morbidity and Mortality Weekly Report. Surveillance Summaries, 57(10), 1–9. 18830214

[vms3586-bib-0005] Beard, C. B. , Occi, J. , Bonilla, D. L. , Egizi, A. M. , Fonseca, D. M. , Mertins, J. W. , Backenson, B. P. , Bajwa, W. I. , Barbarin, A. M. , Bertone, M. A. , Brown, J. , Connally, N. P. , Connell, N. D. , Eisen, R. J. , Falco, R. C. , James, A. M. , Krell, R. K. , Lahmers, K. , Lewis, N. , … Halperin, W. (2018). Multistate infestation with the exotic disease‐vector tick *Haemaphysalis longicornis*—United States, August 2017‐September 2018. MMWR. Morbidity and Mortality Weekly Report, 67(47), 1310–1313. 30496158 10.15585/mmwr.mm6747a3 PMC6276380

[vms3586-bib-0006] Bowman, D. , Little, S. E. , Lorentzen, L. , Shields, J. , Sullivan, M. P. , & Carlin, E. P. (2009). Prevalence and geographic distribution of *Dirofilaria immitis*, *Borrelia burgdorferi*, *Ehrlichia canis*, and *Anaplasma phagocytophilum* in dogs in the United States: results of a national clinic‐based serologic survey. Veterinary Parasitology, 160(1‐2), 138–148. 19150176 10.1016/j.vetpar.2008.10.093

[vms3586-bib-0007] Brownstein, J. S. , Holford, T. R. , & Fish, D. (2005). Effect of climate change on lyme disease risk in North America. EcoHealth, 2(1), 38–46. 19008966 10.1007/s10393-004-0139-x PMC2582486

[vms3586-bib-0008] Chen, X. , Xu, S. , Yu, Z. , Guo, L. , Yang, S. , Liu, L. , Yang, X. , & Liu, J. (2014). Multiple lines of evidence on the genetic relatedness of the parthenogenetic and bisexual *Haemaphysalis longicornis* (Acari: Ixodidae). Infection, Genetics and Evolution: Journal of Molecular Epidemiology and Evolutionary Genetics in Infectious Diseases, 21, 308–314. 10.1016/j.meegid.2013.12.002 24316292

[vms3586-bib-0009] Chomel, B. (2011). Tick‐borne infections in dogs—An emerging infectious threat. Veterinary Parasitology, 179(4), 294–301. 21777730 10.1016/j.vetpar.2011.03.040

[vms3586-bib-0010] Couret, J. , Dyer, M. C. , Mather, T. N. , Han, S. , Tsao, J. I. , Lebrun, R. A. , & Ginsberg, H. S. (2017). Acquisition of *Borrelia burgdorferi* infection by larval *Ixodes scapularis* (Acari: Ixodidae) associated with engorgement measures. Journal of Medical Entomology, 54(4), 1055–1060. 28399208 10.1093/jme/tjx053 PMC11502958

[vms3586-bib-0011] Dahlgren, F. S. , Paddock, C. D. , Springer, Y. P. , Eisen, R. J. , & Behravesh, C. B. (2016). Expanding range of *amblyomma americanum* and simultaneous changes in the epidemiology of spotted fever group rickettsiosis in the United States. American Journal of Tropical Medicine and Hygiene, 94(1), 35–42. 10.4269/ajtmh.15-0580 PMC4710442 26503270

[vms3586-bib-0012] de la Fuente, J. , Villar, M. , Contreras, M. , Moreno‐Cid, J. A. , Merino, O. , Pérez de la Lastra, J. M. , de la Fuente, G. , & Galindo, R. C. (2015). Prospects for vaccination against the ticks of pets and the potential impact on pathogen transmission. Veterinary Parasitology, 208(1‐2), 26–29. 25555312 10.1016/j.vetpar.2014.12.015

[vms3586-bib-0013] de Wet, S. , Rutz, H. , Hinckley, A. F. , Hook, S. A. , Campbell, S. , & Feldman, K. A. (2020). Love the ones you're with: Characteristics and behaviour of Maryland pets and their owners in relation to tick encounters. Zoonoses and Public Health, 67(8), 876–881. 33112510 10.1111/zph.12768

[vms3586-bib-0014] des Vignes, F. , Piesman, J. , Heffernan, R. , Schulze, T. L. , Stafford, K. C. 3rd , & Fish, D. (2001). Effect of tick removal on transmission of *Borrelia burgdorferi* and *Ehrlichia phagocytophila* by *Ixodes scapularis* nymphs. Journal of Infectious Diseases, 183(5), 773–778. 10.1086/318818 11181154

[vms3586-bib-1015] Diuk‐Wasser, M. A. , Hoen, A. G. , Cislo, P. , Brinkerhoff, R. , Hamer, S. A. , Rowland, M. , Cortinas, R. , Vourc'h, G. , Melton, F. , Hickling, G. J. , Tsao, J. I. , Bunikis, J. , Barbour, A. G. , Kitron, U. , Piesman, J. , & Fish, D. (2012). Human risk of infection with Borrelia burgdorferi, the Lyme disease agent, in eastern United States. The American Journal of Tropical Medicine and Hygiene, 86(2), 320–327. 22302869 10.4269/ajtmh.2012.11-0395 PMC3269287

[vms3586-bib-0015] Dolan, M.C. , Breuner, N.E. , Hojgaard, A. , Boegler, K.A. , Hoxmeier, C. , Replogle, A.J. , Eisen, L. (2017). Transmission of the Lyme disease spirochete *Borrelia mayonii* in relation to duration of attachment by nymphal *Ixodes scapularis* (Acari:Ixodidae). Journal of Medical Entomology. 54(5):1360‐1364. 28874016 10.1093/jme/tjx089 PMC5968629

[vms3586-bib-0016] Dryden, M. W. , & Payne, P. A. (2004). Biology and control of ticks infesting dogs and cats in North America. Veterinary Therapeutics: Research in Applied Veterinary Medicine, 5(2), 139–154. Retrieved from https://www.ncbi.nlm.nih.gov/pubmed/15468011 15468011

[vms3586-bib-0017] Duncan, K. T. , Saleh, M.N. , Sundstrom, K.D. , and Little, S.E. (2021). *Dermacentor variabilis* is the predominant *Dermacentor* spp. (Acari: Ixodidae) feeding on dogs and cats throughout the United States. Journal of Medical Entomology, 58, 1241–1247. 33615364 10.1093/jme/tjab007 PMC8122232

[vms3586-bib-0018] Eisen, L. (2018). Pathogen transmission in relation to duration of attachment by *Ixodes scapularis* ticks. Ticks and Tick‐Borne Diseases, 9(3), 535–542. 29398603 10.1016/j.ttbdis.2018.01.002 PMC5857464

[vms3586-bib-0019] Eisen, R. J. , Eisen, L. , & Beard, C. B. (2016). County‐scale distribution of *Ixodes scapularis* and *Ixodes pacificus* (Acari: Ixodidae) in the continental United States. Journal of Medical Entomology, 53(2), 349–386. 26783367 10.1093/jme/tjv237 PMC4844559

[vms3586-bib-0020] Elchos, B. N. , & Goddard, J. (2003). Implications of presumptive fatal Rocky Mountain spotted fever in two dogs and their owner. Journal of the American Veterinary Medical Association, 223(10), 1450–1452, 1433. 14627095 10.2460/javma.2003.223.1450

[vms3586-bib-0021] Eng, T. R. , Wilson, M. L. , Spielman, A. , & Lastavica, C. C. (1988). Greater risk of *Borrelia burgdorferi* infection in dogs than in people. Journal of Infectious Diseases, 158(6), 1410–1411. 10.1093/infdis/158.6.1410 3198950

[vms3586-bib-0022] Falco, R. C. , Daniels, T. J. , Vinci, V. , McKenna, D. , Scavarda, C. , & Wormser, G. P. (2018). Assessment of duration of tick feeding by the scutal index reduces need for antibiotic prophylaxis after *Ixodes scapularis* tick bites. Clinical Infectious Diseases: An Official Publication of the Infectious Diseases Society of America, 67(4), 614–616. 29579163 10.1093/cid/ciy221

[vms3586-bib-0023] Fernandez, M. P. , Bron, G. M. , Kache, P. A. , Larson, S. R. , Maus, A. , Gustafson, D. Jr , Tsao, J. I. , Bartholomay, L. C. , Paskewitz, S. M. , & Diuk‐Wasser, M. A. (2019). Usability and feasibility of a smartphone app to assess human behavioral factors associated with tick exposure (The Tick App): Quantitative and qualitative study. JMIR mHealth and uHealth, 7(10), e14769. 31651409 10.2196/14769 PMC6913724

[vms3586-bib-0024] Fischhoff, I. R. , Keesing, F. , Pendleton, J. , DePietro, D. , Teator, M. , Duerr, S. T. K. , Mowry, S. , Pfister, A. , LaDeau, S. L. , & Ostfeld, R. S. (2019). Assessing effectiveness of recommended residential yard management measures against ticks. Journal of Medical Entomology, 56, 1420–1427. 31120510 10.1093/jme/tjz077 PMC6736118

[vms3586-bib-0025] Fritz, C. L. (2009). Emerging tick‐borne diseases. The Veterinary Clinics of North America. Small Animal Practice, 39(2), 265–278. 19185193 10.1016/j.cvsm.2008.10.019

[vms3586-bib-1025] Ghosh, P. , Saleh, M. N. , Sundstrom, K. D. , Ientile, M. , & Little, S. E. (2021). Ixodes spp. from Dogs and Cats in the United States: Diversity, Seasonality, and Prevalence of Borrelia burgdorferi and Anaplasma phagocytophilum. Vector Borne and Zoonotic Diseases, 21(1), 11–19. 32986535 10.1089/vbz.2020.2637 PMC9469741

[vms3586-bib-0026] Gilliam, M. E. , Rechkemmer, W. T. , McCravy, K. W. , & Jenkins, S. E. (2018). The influence of prescribed fire, habitat, and weather on *Amblyomma americanum* (Ixodida: Ixodidae) in West‐Central Illinois, USA. Insects, 9(2), 36. 10.3390/insects9020036 PMC6023455 29565805

[vms3586-bib-0027] Ginsberg, H. S. , & Zhioua, E. (1999). Influence of deer abundance on the abundance of questing adult *Ixodes scapularis* (Acari: Ixodidae). Journal of Medical Entomology, 36(3), 376–381. 10337111 10.1093/jmedent/36.3.376

[vms3586-bib-0028] Guernier, V. , Milinovich, G. J. , Bezerra Santos, M. A. , Haworth, M. , Coleman, G. , & Soares Magalhaes, R. J. (2016). Use of big data in the surveillance of veterinary diseases: Early detection of tick paralysis in companion animals. Parasites & Vectors, 9(1), 303. 27215214 10.1186/s13071-016-1590-6 PMC4877981

[vms3586-bib-0029] Guerra, M. , Walker, E. , Jones, C. , Paskewitz, S. , Cortinas, M. R. , Stancil, A. , Beck, L. , Bobo, M. , & Kitron, U. (2002). Predicting the risk of Lyme disease: Habitat suitability for *Ixodes scapularis* in the north central United States. Emerging Infectious Diseases, 8(3), 289–297. 11927027 10.3201/eid0803.010166 PMC2732460

[vms3586-bib-0030] Guerra, M. A. , Walker, E. D. , & Kitron, U. (2001). Canine surveillance system for Lyme borreliosis in Wisconsin and northern Illinois: Geographic distribution and risk factor analysis. American Journal of Tropical Medicine and Hygiene, 65(5), 546–552. 10.4269/ajtmh.2001.65.546 11716112

[vms3586-bib-0031] Hall, B. , Motzkin, G. , Foster, D. R. , Syfert, M. , & Burk, J. (2002). Three hundred years of forest and land‐use change in Massachusetts, USA. Journal of Biogeography, 29(10‐11), 1319–1335.

[vms3586-bib-0032] Halperin JJ , Baker P , Wormser GP. (2013). Common misconceptions about Lyme disease. American Journal of Medicine, 126(3):264 10.1016/j.amjmed.2012.10.008 23321431

[vms3586-bib-0033] Hansford, K. M. , Pietzsch, M. E. , Cull, B. , Gillingham, E. L. , & Medlock, J. M. (2018). Potential risk posed by the importation of ticks into the UK on animals: records from the Tick Surveillance Scheme. Veterinary Record, 182(4), 107. 10.1136/vr.104263 29217768

[vms3586-bib-0034] Hook, S. A. , Nelson, C. A. , & Mead, P. S. (2015). U.S. public's experience with ticks and tick‐borne diseases: Results from national HealthStyles surveys. Ticks and Tick‐Borne Diseases, 6(4), 483–488. 25887156 10.1016/j.ttbdis.2015.03.017 PMC7053299

[vms3586-bib-0035] Isman, M. B. , & Grieneisen, M. L. (2014). Botanical insecticide research: Many publications, limited useful data. Trends in Plant Science, 19(3), 140–145. 24332226 10.1016/j.tplants.2013.11.005

[vms3586-bib-0036] Johnson, J. L. , Ginsberg, H. S. , Zhioua, E. , Whitworth, U. G. Jr , Markowski, D. , Hyland, K. E. , & Hu, R. (2004). Passive tick surveillance, dog seropositivity, and incidence of human Lyme disease. Vectorborne and Zoonotic Diseases, 4(2), 137–142. 10.1089/1530366041210710 15228814

[vms3586-bib-0037] Johnson, K. A. (2018). Complementary and alternative veterinary medicine: Where things stand for feline health. Science & Technology Libraries, 37(4), 338–376.

[vms3586-bib-0038] Jones, E. H. , Hinckley, A. F. , Hook, S. A. , Meek, J. I. , Backenson, B. , Kugeler, K. J. , & Feldman, K. A. (2018). Pet ownership increases human risk of encountering ticks. Zoonoses and Public Health, 65(1), 74–79. 28631423 10.1111/zph.12369 PMC7053298

[vms3586-bib-0039] Jones, T. F. , Garman, R. L. , LaFleur, B. , Stephan, S. J. , & Schaffner, W. (2002). Risk factors for tick exposure and suboptimal adherence to preventive recommendations. American Journal of Preventive Medicine, 23(1), 47–50. 12093423 10.1016/s0749-3797(02)00440-3

[vms3586-bib-0040] Kidd, L. , & Breitschwerdt, E. B. (2003). Transmission times and prevention of tick‐borne diseases in dogs. Compendium, 25(10), 742–751.

[vms3586-bib-0041] Koffi, J. K. , Savage, J. , Thivierge, K. , Lindsay, L. R. , Bouchard, C. , Pelcat, Y. , & Ogden, N. H. (2017). Evaluating the submission of digital images as a method of surveillance for *Ixodes scapularis* ticks. Parasitology, 144(7), 877–883. 28345501 10.1017/S0031182017000117 PMC5471820

[vms3586-bib-0042] Kopsco, H. L. , Duhaime, R. J. , & Mather, T. N. (2021). Assessing public tick identification ability and tick bite riskiness using passive photograph‐based crowdsourced tick surveillance. Journal of Medical Entomology, 58(2), 837–846. 33146378 10.1093/jme/tjaa196

[vms3586-bib-0043] Kopsco, H. L. , Xu, G. , Luo, C.‐Y. , Rich, S. M. , & Mather, T. N. (2020). Crowdsourced photographs as an effective method for large‐scale passive tick surveillance. Journal of Medical Entomology, 57(6), 1955–1963. 32812635 10.1093/jme/tjaa140

[vms3586-bib-1043] Lyme Disease (2020). Centers for Disease Control and Prevention, National Center for Emerging and Zoonotic Infectious Diseases (NCEZID), Division of Vector‐Borne Diseases (DVBD). Retrieved from https://www.cdc.gov/ticks/tickbornediseases/lyme.html.

[vms3586-bib-0044] Lindenmayer, J. M. , Marshall, D. , & Onderdonk, A. B. (1991). Dogs as sentinels for Lyme disease in Massachusetts. American Journal of Public Health, 81(11), 1448–1455. 1951802 10.2105/ajph.81.11.1448 PMC1405676

[vms3586-bib-0045] Little, S. E. , Barrett, A. W. , Nagamori, Y. , Herrin, B. H. , Normile, D. , Heaney, K. , & Armstrong, R. (2018). Ticks from cats in the United States: Patterns of infestation and infection with pathogens. Veterinary Parasitology, 257, 15–20. 29907187 10.1016/j.vetpar.2018.05.002

[vms3586-bib-0046] Little, S. E. , Beall, M. J. , Bowman, D. D. , Chandrashekar, R. , & Stamaris, J. (2014). Canine infection with *Dirofilaria immitis*, *Borrelia burgdorferi*, *Anaplasma* spp., and *Ehrlichia* spp. in the United States, 2010–2012. Parasites & Vectors, 7, 257. 24886589 10.1186/1756-3305-7-257 PMC4057565

[vms3586-bib-0047] Littman, M. P. , Goldstein, R. E. , Labato, M. A. , Lappin, M. R. , & Moore, G. E. (2018). ACVIM Small animal consensus statement on lyme disease in dogs: Diagnosis, treatment, and prevention. Journal of Veterinary Internal Medicine/American College of Veterinary Internal Medicine, 20(2), 422–434. 10.1892/0891-6640(2006)20[422:asacso]2.0.co;2 16594606

[vms3586-bib-0049] Mather, T.N. , Fish, D. , & Coughlin, R.T. (1994). Competence of dogs as reservoirs for Lyme disease spirochetes (*Borrelia burgdorferi*). JAVMA, 205, 186–188. 7928571

[vms3586-bib-0050] Nagamori, Y. , & Reichard, M. V. (2015). Feline tick‐borne diseases. Today Veterinary Practice, 3, 69–74.

[vms3586-bib-0051] Nieto, N. C. , Porter, W. T. , Wachara, J. C. , Lowrey, T. J. , Martin, L. , Motyka, P. J. , & Salkeld, D. J. (2018). Using citizen science to describe the prevalence and distribution of tick bite and exposure to tick‐borne diseases in the United States. PloS One, 13(7), e0199644. 30001350 10.1371/journal.pone.0199644 PMC6042714

[vms3586-bib-0052] Ogden, N. H. , & Lindsay, L. R. (2016). Effects of climate and climate change on vectors and vector‐borne diseases: Ticks are different. Trends in Parasitology, 32(8), 646–656. 27260548 10.1016/j.pt.2016.04.015

[vms3586-bib-0053] Ogden, N. H. , Radojevic, M. , Wu, X. , Duvvuri, V. R. , Leighton, P. A. , & Wu, J. (2014). Estimated effects of projected climate change on the basic reproductive number of the Lyme disease vector *Ixodes scapularis* . Environmental Health Perspectives, 122(6), 631–638. 24627295 10.1289/ehp.1307799 PMC4050516

[vms3586-bib-0054] Ostfeld, R. S. , & Brunner, J. L. (2015). Climate change and *Ixodes* tick‐borne diseases of humans. Philosophical Transactions of the Royal Society of London. Series B, Biological Sciences, 370(1665), 20140051. 25688022 10.1098/rstb.2014.0051 PMC4342967

[vms3586-bib-0055] Piesman, J. , Mather, T.N. , Sinsky, R.J. , & Spielman, A. (1987). Duration of tick attachment and *Borrelia burgdorferi* transmission. Journal of Clinical Microbiology, 25(3), 557. 3571459 10.1128/jcm.25.3.557-558.1987 PMC265989

[vms3586-bib-0056] Pollock, N. B. , Gawne, E. , & Taylor, E. N. (2015). Effects of temperature on feeding duration, success, and efficiency of larval western black‐legged ticks (Acari: Ixodidae) on western fence lizards. Experimental & Applied Acarology, 67(2), 299–307. 26188858 10.1007/s10493-015-9950-z

[vms3586-bib-0057] Rainey, T. , Occi, J. L. , Robbins, R. G. , & Egizi, A. (2018). Discovery of *Haemaphysalis longicornis* (Ixodida: Ixodidae) parasitizing a sheep in New Jersey, United States. Journal of Medical Entomology, 55, 757–759. 29471482 10.1093/jme/tjy006

[vms3586-bib-1058] Rand, P. W. , Lubelczyk, C. , Lavigne, G. R. , Elias, S. , Holman, M. S. , Lacombe, E. H. , & Smith, R. P. , Jr. (2003). Deer density and the abundance of Ixodes scapularis (Acari: Ixodidae). Journal of Medical Entomology, 40(2), 179–184. 12693846 10.1603/0022-2585-40.2.179

[vms3586-bib-0059] Rea, L. M. , & Parker, R. A. (1992). Designing and conducting survey research: a comprehensive guide. San Francisco: Jossey‐Bass Publishers.

[vms3586-bib-0060] Reichard MV , Edwards AC , Meinkoth JH , Snider, T. A. , Meinkoth, K. R. , Heinz, R. E. , & Little, S. E. (2010). Confirmation of *Amblyomma americanum* (Acari: Ixodidae) as a vector for *Cytauxzoon felis* (Piroplasmorida: Theileriidae) to domestic cats. Journal of Medical Entomology, 47, 890–896. 20939386 10.1603/me10013

[vms3586-bib-0061] Rosenberg, R. , Lindsey, N. P. , Fischer, M. , Gregory, C. J. , Hinckley, A. F. , Mead, P. S. , Paz‐Bailey, G. , Waterman, S. H. , Drexler, N. A. , Kersh, G. J. , Hooks, H. , Partridge, S. K. , Visser, S. N. , Beard, C. B. , & Petersen, L. R. (2018). *Vital signs*: Trends in reported vectorborne disease cases—United States and Territories, 2004–2016. MMWR. Morbidity and Mortality Weekly Report, 67(17), 496–501. 29723166 10.15585/mmwr.mm6717e1 PMC5933869

[vms3586-bib-0062] Saleh, M. N. , Sundstrom, K. D. , Duncan, K. T. , Ientile, M. M. , Jordy, J. , Ghosh, P. , & Little, S. E. (2019). Show us your ticks: a survey of ticks infesting dogs and cats across the USA. Parasites & Vectors, 12(1), 595. 31856893 10.1186/s13071-019-3847-3 PMC6923977

[vms3586-bib-0063] Salkeld, D. J. , Nieto, N. C. , Carbajales‐Dale, P. , Carbajales‐Dale, M. , Cinkovich, S. S. , & Lambin, E. F. (2015). Disease risk & landscape attributes of tick‐borne borrelia pathogens in the San Francisco Bay Area, California. PloS One, 10(8), e0134812. 26288371 10.1371/journal.pone.0134812 PMC4545583

[vms3586-bib-0064] Sánchez‐Vizcaíno, F. , Wardeh, M. , Heayns, B. , Singleton, D. A. , Tulloch, J. S. P. , McGinley, L. , Newman, J. , Noble, P. J. , Day, M. J. , Jones, P. H. , & Radford, A. D. (2016). Canine babesiosis and tick activity monitored using companion animal electronic health records in the UK. Veterinary Record, 179(14), 358. 10.1136/vr.103908 PMC5099196 27484328

[vms3586-bib-0065] Schwartz, A. M. , Hinckley, A. F. , Mead, P. S. , Hook, S. A. , & Kugeler, K. J. (2017). Surveillance for Lyme Disease—United States, 2008–2015. Morbidity and Mortality Weekly Report. Surveillance Summaries, 66(22), 1–12. 10.15585/mmw.ss6622a1 PMC5829628 29120995

[vms3586-bib-0066] Shannon, A. B. , Rucinsky, R. , Gaff, H. D. , & Brinkerhoff, R. J. (2017). *Borrelia miyamotoi*, Other vector‐borne agents in cat blood and ticks in Eastern Maryland. EcoHealth, 14(4), 816–820. 28879619 10.1007/s10393-017-1268-3

[vms3586-bib-0067] Shaw, S. E. , Day, M. J. , Birtles, R. J. , & Breitschwerdt, E. B. (2001). Tick‐borne infectious diseases of dogs. Trends in Parasitology, 17(2), 74–80. 11228013 10.1016/s1471-4922(00)01856-0

[vms3586-bib-0068] Sonenshine, D. E. (2018). Range expansion of tick disease vectors in North America: Implications for spread of tick‐borne disease. International Journal of Environmental Research and Public Health, 15(3), 478. 10.3390/ijerph15030478 PMC5877023 29522469

[vms3586-bib-0069] Sood, S.K. , Salzman, M.B. , Johnson, B.J.B. , Happ, C.M. , Feig, K. , Carmody, L. , Rubin, L.G. , Hilton, E. , Piesman, J. (1997). Duration of tick attachment as a predictor of the risk of Lyme disease in an area in which Lyme disease is endemic. Journal of Infectious Diseases, 175, 996‐999. 10.1086/514009 9086168

[vms3586-bib-0070] Springer, Y. P. , Eisen, L. , Beati, L. , James, A. M. , & Eisen, R. J. (2014). Spatial distribution of counties in the continental United States with records of occurrence of *Amblyomma americanum* (Ixodida: Ixodidae). Journal of Medical Entomology, 51(2), 342–351. 24724282 10.1603/me13115 PMC4623429

[vms3586-bib-0071] Tahir, D. , Meyer, L. , Fourie, J. , Jongejan, F. , Mather, T. , Choumet, V. , Blagburn, B. , Straubinger, R.K. , Varloud, M. (2020). Interrupted blood feeding in ticks: causes and consequences. Microorganisms, 8(6), 910. 10.3390/microorganisms8060910 PMC7355616 32560202

[vms3586-bib-0072] Tran, P. M. , & Waller, L. (2013). Effects of landscape fragmentation and climate on Lyme disease incidence in the northeastern United States. EcoHealth, 10(4), 394–404. 24419663 10.1007/s10393-013-0890-y

[vms3586-bib-0073] Tulloch, J. S. P. , McGinley, L. , Sánchez‐Vizcaíno, F. , Medlock, J. M. , & Radford, A. D. (2017). The passive surveillance of ticks using companion animal electronic health records. Epidemiology and Infection, 145(10), 2020–2029. 28462753 10.1017/S0950268817000826 PMC5968307

[vms3586-bib-0074] Tulloch, J. S. P. , Vivancos, R. , Christley, R. M. , Radford, A. D. , & Warner, J. C. (2019). Mapping tweets to a known disease epidemiology: A case study of Lyme disease in the United Kingdom and Republic of Ireland. Journal of Biomedical Informatics: X, 4, 100060. 10.1016/j.yjbinx.2019.100060 34384577

[vms3586-bib-0075] Wagner, B. , & Erb, H. N. (2012). Dogs and horses with antibodies to outer‐surface protein C as on‐time sentinels for ticks infected with *Borrelia burgdorferi* in New York State in 2011. Preventive Veterinary Medicine, 107(3‐4), 275–279. 22841496 10.1016/j.prevetmed.2012.07.002

[vms3586-bib-0076] White, A. , & Gaff, H. (2018). Review: Application of tick control technologies for blacklegged, lone star, and american dog ticks. Journal of Integrated Pest Management, 9(1), 12.

[vms3586-bib-0077] Xu, G. , Mather, T. N. , Hollingsworth, C. S. , & Rich, S. M. (2016). Passive Surveillance of *Ixodes scapularis* (Say), their biting activity, and associated pathogens in Massachusetts. Vector‐Borne and Zoonotic Diseases, 16(8), 520–527. 27248292 10.1089/vbz.2015.1912 PMC4960492

[vms3586-bib-0078] Yeh, M. T. , Bak, J. M. , Hu, R. , Nicholson, M. C. , Kelly, C. , & Mather, T. N. (1995). Determining the duration of *Ixodes scapularis* (Acari: Ixodidae) attachment to tick‐bite victims. Journal of Medical Entomology, 32(6), 853–858. 8551509 10.1093/jmedent/32.6.853

[vms3586-bib-0079] Zeimes, C. B. , Olsson, G. E. , Hjertqvist, M. , & Vanwambeke, S. O. (2014). Shaping zoonosis risk: landscape ecology vs. landscape attractiveness for people, the case of tick‐borne encephalitis in Sweden. Parasites & Vectors, 7(1), 370. 25128197 10.1186/1756-3305-7-370 PMC4143547

